# Sex Differences in Outcome and Mortality in Urinary Tract Infections in the Emergency Department: The UTILY Study

**DOI:** 10.5811/westjem.48903

**Published:** 2026-10-19

**Authors:** Carmine Nasta, Lorenzo Onorato, Enrico Allegorico, Francesca Palumbo, Biagio Migliaccio, Adelaide Mariniello, Federica Esposito, Alessandro Manzoni, Antonio Voza, Margherita Macera, Caterina Monari, Romeo Giordano, Fabio Giuliano Numis, Giovanna Guiotto, Rosa Raucci, Ferdinando Dello Vicario, Rodolfo Nasti, Mauro Giordano, Nicola Coppola, UTILY study group, Lorenzo Onorato, Margherita Macera, Caterina Monari, Ilaria De Luca, Annabella Salvati, Alessandro Cornelli, Nicola Coppola, Fabio Giuliano Numis, Enrico Allegorico, Monica Vanni, Piero Bianco, Stefano Aiello, Antonio Carannante, Nicola Compagnone, Carmen Rapuano, Marina D’Isanto, Biagio Migliaccio, Nicoletta Vitelli, Francesca Delgado, Giovanna Guiotto, Carmine Nasta, Romeo Morelli, Francesca Palumbo, Alfredo Palumbo, Mauro Giordano, Maria Teresa Florio, Anna Amato, Anna Santagata, Adelaide Mariniello, Nicola Quaranta, Vincenza Serrao, Augusto Delle Femine, Ilaria Guida, Annalisa Amelia, Federica Miglietta, Rosa Raucci, Roberta Sciorio, Kim Russo, Federica Esposito, Filomena Fabozzi, Luca De Capua, Gennaro Maria Borrelli, Rosa Giamattei, Raffaella Coppola, Rodolfo Nasti, Evaluna Perez Guillen, Antonio Voza

**Affiliations:** * University of Campania “L. Vanvitell,” Naples, Department of Advanced Medical and Surgical Sciences, Department of Emergency Medicine, Marcianise (Caserta), Italy; † University of Campania Luigi Vanvitelli, Department of Mental Health and Public Medicine, Section of Infectious Diseases, Naples, Italy; ‡ Ospedale Santa Maria delle Grazie, ASL Napoli 2 Nord, Unità Operativa Complessa Medicina d’Emergenza ed Urgenza, Pozzuoli (Naples), Italy; § Ospedale del Mare, ASL Napoli 1 Centro, Unità Operativa Complessa Medicina di Emergenza-Urgenza, Naples, Italy; ¶ Ospedale Betania, Pronto Soccorso, Napoli, Italy; || Pronto Soccorso, P.O. Moscati Aversa (Caserta), Italy; # Ospedale San Giuliano, Asl Napoli 2 Nord, Unità Operativa Semplice Dipartimentale Pronto Soccorso-Osservazione Breve Intensiva, Giugliano (NA), Italy; ** IRCCS Humanitas Research Hospital, Department of Biomedical Sciences, Milan, Italy

## Abstract

**Introduction:**

In urinary tract infections (UTI) sex-related differences in comorbidities, antimicrobial resistance, and outcomes have been described, but it remains unclear whether these disparities reflect sex-related biological mechanisms or are primarily explained by age and comorbidity, especially in emergency settings. Our objective was to evaluate sex-related differences in microbiology, antimicrobial resistance, and outcomes among adults with potentially multidrug-resistant UTIs presenting to the emergency department (ED), and to determine whether sex independently predicts multidrug-resistant infections and outcomes after adjustment for age and comorbidity.

**Methods:**

We conducted a prospective multicenter study enrolling all adult patients with suspected or confirmed UTIs admitted to one of the eight EDs involved from February 2023–July 2024. Our primary outcome was the prevalence of multidrug-resistant UTIs and whether sex independently predicted multidrug-resistant infection after adjustment for age and comorbidity. Secondary outcomes included hospitalization, adequacy of empirical antibiotic therapy, clinical response at 7 days, and 7-day and 30-day all-cause mortality. Demographic, clinical, and microbiological data were compared by sex. To account for baseline differences in age and comorbidity burden (Charlson Comorbidity Index), multivariable logistic regression models were applied to identify independent predictors of multidrug-resistant infection, hospitalization, and 30-day mortality.

**Results:**

We included 679 patients (358 males, 321 females). Mean age was 70.1 years (standard deviation 17.9) in males and 63.1 years (21.4) in females (*P* < .001). The Charlson Comorbidity Index was higher in males (2.50 [2.45]) than in females (1.85 [2.32]) (*P* = .01). Multidrug-resistant infection occurred in 107/321 females (33.5%) and 166/358 males (46.5%) (*P* = .02). Adequate empirical therapy was achieved in 213/321 females (66.2%) and 221/358 males (61.8%) (*P* = .10). Hospitalization occurred in 176/321 females (54.9%) and 175/358 males (48.8%) (*P* = .09). Seven-day mortality was 14/321 females (4.5%) and 28/358 males (7.9%) (*P* = .05), while 30-day mortality was 32/321 females (9.9%) and 50/358 males (14.0%) (*P* = .05). After adjustment for age and comorbidity, male sex remained independently associated with multidrug-resistant infection (adjusted odds ratio 1.45; 95% CI, 1.03–2.10, *P* sex = .02; *P* age = .01).

**Conclusion:**

Male sex was independently associated with multidrug-resistant urinary tract infections in the emergency department, even after adjustment for age and comorbidity. These findings highlight the need for sex- and risk-based approaches to empirical therapy and antimicrobial stewardship in acute care settings.

## INTRODUCTION

Urinary tract infections (UTI) are among the leading infectious causes of emergency department (ED) visits worldwide, representing a major burden for acute care services.^1^,^2^ While women experience a higher incidence of UTIs overall, men more often present with complicated infections, greater comorbidity burden, and higher rates of multidrug-resistant pathogens.^2^–^5^ Despite these recognized differences, evidence describing sex-specific clinical characteristics, microbiological profiles, and short-term outcomes in the ED setting remains limited—particularly in real-world multicenter cohorts. Moreover, the observed disparities between sexes may be at least partly driven by age and comorbidity, as male patients typically present at an older age and with higher Charlson Comorbidity Index (CCI) scores. These confounding factors complicate the interpretation of sex-related differences in infection severity, antimicrobial resistance, and outcomes.

Understanding whether sex independently influences multidrug-resistance risk and clinical outcomes, beyond the effects of age and comorbidity, is crucial to guide empirical antibiotic selection and improve antimicrobial stewardship in emergency care.

In this multicenter observational study we aimed to evaluate sex-related differences in clinical presentation, microbiological findings, antimicrobial resistance, and outcomes among adults with suspected or confirmed UTI presenting to eight EDs in Italy, while adjusting for age and comorbidity burden to identify independent predictors of multidrug-resistant infection and adverse outcomes.

## METHODS

We conducted this prospective, multicenter observational study across eight EDs participating in the UTI in Italy (UTILY) study from February 2023–July 2024. Five of the eight healthcare facilities were in Naples; two in Caserta, Campania, in Southern Italy; and one in Milan, Lombardy, in Northern Italy. We included all adult patients (≥ 18 years of age) presenting to the ED with a clinical or microbiological diagnosis of UTI, or with suspected UTI based on symptoms and laboratory findings. Suspected UTI was defined as the presence of at least one urinary symptom(dysuria, urinary frequency, urgency, suprapubic pain, flank pain) or fever without an alternative source, in combination with lab findings suggestive of infection (positive urinalysis including leukocyturia and/or nitrites). We defined a urine culture growing a potential uropathogen at counts ≥ 105 colony-forming units/mL as positive. We excluded from the analysis cultures growing a mixed flora, non-speciated *Streptococcus* or *Staphylococcus* organisms or *Candida* species.

Patients presenting with suggestive symptoms and positive urinalysis but with pending urine culture results at ED admission were initially classified as suspected UTI and were included in the study, with final classification based on microbiological results when available.

**Population Health Research Capsule**

What do we already know about this issue?

*Sex differences in urinary tract infections (UTI) exist, with men showing higher multidrug-resistance rates and worse outcomes.*

What was the research question?

*Does sex independently predict multidrug-resistant UTI and outcomes after adjusting for age and comorbidity in emergency department patients?*

What was the major finding of the study?

*Male sex predicted multidrug-resistant UTI (aOR 1.45; 95% CI, 1.03–2.10; P = .04; 46.5% vs 33.5%).*

How does this improve population health?

*Identifying male sex as a multidrug-resistant risk factor supports targeted treatment and stewardship, thereby improving outcomes and reducing inappropriate antibiotic use.*

We retrieved information on prior microbiological diagnosis from electronic health records, referral documentation, or previous lab reports. Patients were excluded if they were < 18 years of age, had a negative urine culture, or declined to participate in the study. In all cases, decisions regarding hospital admission and the choice of the empirical treatment were made by the treating physician, according to local and international guidelines. For all enrolled patients, we collected the following information using a predefined data collection form: age; sex; and comorbidity. We calculated the CCI based on comorbid conditions only, excluding the age component, and used it as a measure of comorbidity burden. Age was analyzed separately as an independent variable in the regression models.^6^ Additional data collected included the following: hospitalization and antimicrobial treatment during the previous 90 days; invasive procedures in the previous 30 days; presence of devices (urinary catheter, ureteral stent, nephrostomy); any ongoing antibiotic therapy at the time of urine sample collection; severity of infection as per the Sequential Organ Failure Assessment score; presence of sepsis/septic shock; empiric antibiotic therapy; duration of treatment; hospital admission; microorganism isolated and antimicrobial susceptibility profile; clinical response at 7 days; and mortality at 7 and 30 days. Patients with incomplete records or missing outcome data were excluded. Microbiological data included pathogen identification and antimicrobial susceptibility testing, which were used to define multidrug-resistant status. Therapeutic data encompassed both empirical and targeted antibiotic regimens.

The primary outcome was multidrug-resistant infection, defined as the isolation of a pathogen resistant to at least one agent in three or more antimicrobial classes. Secondary outcomes included hospitalization, clinical response at 7 days, and all-cause mortality at 7- and 30-days following ED presentation.

We summarized continuous variables as means with standard deviations or medians with interquartile ranges and compared them using the Mann–Whitney U test. Categorical variables were expressed as counts and percentages and compared using the chi-square or Fisher exact test, as appropriate. To account for potential confounding by age and comorbidity burden, we performed. multivariable logistic regression models to identify independent predictors of multidrug-resistant infection, hospitalization, and 30-day mortality. Covariates included sex, age, and CCI. Results were expressed as adjusted odds ratios (OR) with 95% confidence intervals. Model performance was evaluated using the McFadden pseudo-*R*^2^, Akaike information criterion, and Bayesian information criterion. We performed statistical analyses using IBM SPSS Statistics v28.0 (International Business Machines Corp., Armonk, NY) and R version 4.3.2 (The R Foundation for Statistical Computing, Vienna, Austria).

The study was approved by the ethics committee of the University of Campania “L. Vanvitelli,” Naples (No. 35,093/2023), and conducted in accordance with the principles of the Declaration of Helsinki and the Strengthening the Reporting of Observational studies in Epidemiology (STROBE) guidelines for observational studies.

### Definitions

The diagnoses of UTI were made in accordance with the guidelines of the European Association of Urology.^7^ The term multidrug resistance was applied according to the definition proposed by Magiorakos et al as acquired nonsusceptibility to at least one agent in three or more antimicrobial categories.^8^ The empirical treatment was defined as appropriate if it showed in vitro activity against the isolated pathogen.

## RESULTS

### Demographics and Comorbidities

A total of 679 patients were included in the study, comprising 358 males (52.7%) and 321 females (47.3%). The overall mean age was 65.7 years (19.1). The comorbidity burden was assessed using the CCI, a validated scoring system that quantifies mortality risk based on weighted chronic conditions. In this cohort, CCI values ranged from 0–18. The maximum possible CCI severity score is 35 points. Male patients were significantly older than females and exhibited a higher comorbidity burden, as reflected by the CCI (2.50 (2.45) vs 1.85 (2.32); *P* = .01).

When stratified by age group, both younger (< 65 years of age) and older men (≥ 65) maintained higher CCI scores compared with their female counterparts, indicating a consistently greater prevalence of chronic conditions among male patients across all age strata.

Notably, rates of multidrug-resistant infection and 30-day mortality were consistently higher in males and older patients, highlighting the combined influence of these factors on adverse outcomes. Continuous variables were analyzed using the Mann–Whitney U test and categorical variables with the chi-square or Fisher exact test.[Table t1-wjem-27-1623]

### Severity of Infection

Overall, 243 patients (35.7%) met sepsis criteria, and 55 (8.1%) had septic shock at presentation. Severe infection (sepsis or septic shock) was significantly more frequent among males than females (50.4% vs 37.7%; *P* = .02), paralleling their higher comorbidity burden. Among patients with septic shock, males exhibited a higher 30-day mortality, although the difference did not reach statistical significance.

### Microbiology and Resistance

Among patients with available microbiological data, multidrug-resistant organisms were isolated more frequently in males than in females (46.5% vs 33.5%; *P* = .02). *Klebsiella spp* and *Enterococcus spp* were more commonly identified in men, whereas *Escherichia Coli* predominated among women. This distribution pattern was consistent across both age strata (< 65 and ≥ 65 years of age), suggesting that the higher multidrug-resistant rates observed in males were not solely age dependent. 14 Detailed pathogen distribution and antimicrobial resistance profiles are reported in Table 2 and illustrated in Figure 1.

### Therapy and Outcomes

Adequate empirical therapy was achieved in 63% of patients overall, with no significant differences between sexes (61.8% in males vs 66.2% in females; *P* =.10). These findings are consistent with the results of the UTILY antimicrobial stewardship initiative, recently reported by Macera et al (in press), which demonstrated improved antibiotic selection in ED settings through a persuasive stewardship approach.^15^

Approximately 50% of the patients required hospitalization, with comparable rates between men and women (48.8% vs 54.9%; *P* =.09). Mortality rates, however, were higher among males, both at 7 days (7.9% versus 4.5%) and 30 days (14.0% versus 9.9%; *P* = .05). The sex-related gap in short-term outcomes persisted within the ≥ 65 years of age subgroup, in which men also exhibited a greater burden of comorbidities and a higher prevalence of multidrug-resistant infections. These findings suggest that the combined impact of comorbidity and multidrug-resistant infection may partially account for the worse outcomes observed in male patients.

Older adults (≥ 65 years of age) showed markedly higher rates of hospitalization, multidrug-resistant infection, and mortality compared with younger patients. Across both age strata, males exhibited a greater prevalence of multidrug-resistant pathogens, higher short-term (7-day) and 30-day mortality, and slightly lower adequacy of empirical antibiotic therapy than females, despite comparable hospitalization rates. These differences were most pronounced among older men (≥ 65 years of age), who also had the highest comorbidity burden. In multivariable logistic regression, male sex (OR ≈ 1.6; 95% CI, 1.1–2.4) and higher CCI independently predicted multidrug-resistant infection, whereas older age and comorbidity burden were strongly associated with 30-day mortality.

Overall, these findings underscore the synergistic influence of sex and age on infection severity, resistance patterns, and treatment response in patients presenting with UTIs to the ED. We performed a secondary multivariable analysis including sepsis and septic shock at presentation to further explore whether disease severity influenced these associations. When disease severity (sepsis or septic shock) was included in the regression model, male sex remained independently associated with multidrug-resistant infection (adjusted OR 1.38; 95% CI, 1.01–1.95; *P* = .04), while sepsis/septic shock at presentation independently predicted 30-day mortality (adjusted OR 3.20; 95% CI, 1.90–5.35; *P* < .001). The inclusion of severity in the model slightly attenuated the effect size of sex on mortality, suggesting that part of the observed sex difference may be mediated by higher infection severity among males.

Key findings show that older age and male sex are independently associated with worse outcomes, including significantly higher hospitalization rates, multidrug-resistant infection, and 7- and 30-day mortality. Notably, males > 65 years of age had the worst outcomes and the lowest rate of adequate empirical therapy, while clinical response was significantly lower overall in males.

### Predictors of Multidrug-Resistant Infection

In the multivariable logistic regression model adjusted for age and comorbidity burden, male sex remained an independent predictor of multidrug-resistant UTI (adjusted OR 1.45; 95% CI, 1.03–2.10; ***P*** = .04). This association persisted despite adjustment for age and CCI, suggesting that male sex contributes to the risk of antimicrobial resistance beyond baseline clinical differences.^16^

Although older age (≥ 65 years) was also associated with a higher prevalence of multidrug-resistant isolates (OR 1.50; 95% CI, 1.0–2.3), its effect appeared partly mediated by the greater comorbidity burden observed in elderly patients.

A higher CCI showed a borderline association with multidrug-resistant infection (*P* = .06), consistent with the cumulative impact of chronic conditions on infection complexity and resistance selection. No formal interaction between sex and age was detected; however, the combination of male sex and advanced age identified a subgroup of patients at particularly elevated risk for multidrug-resistant infection. These findings highlight that sex, age, and comorbidity act as interrelated but distinct determinants of antimicrobial resistance in the emergency setting. Recognition of these factors may support risk stratification at ED presentation, helping clinicians identify patients at higher risk of multidrug-resistant infection.

Rather than implying systematic use of broader spectrum antibiotics, these findings should be interpreted in the context of clinical severity, prior antibiotic exposure, and local resistance patterns, to support individualized empirical therapy and antimicrobial stewardship strategies.

## DISCUSSION

This multicenter study demonstrates that both sex and age significantly influence the risk of multidrug-resistant UTIs and the clinical presentations in the ED, as well as associated clinical outcomes. Male patients were generally older, had a higher comorbidity burden, and exhibited a greater prevalence of multidrug-resistant pathogens compared with females,^1^–^4^ particularly among those ≥ 65 years of age. These findings are consistent with multicenter ED cohorts and population datasets showing higher multidrug-resistance rates in men and in older adults and support male sex as an independent risk factor for multidrug-resistant infection, even after adjustment for age and comorbidity burden (adjusted OR 1.45; 95% CI, 1.03–2.10).^9^,^10^,^14^

The observed sex differences likely reflect a complex interplay of biological, behavioral, and clinical factors. Male patients more frequently present with complicated infections, prior antibiotic exposure, and structural urinary abnormalities, which may contribute to resistance selection. The association between older age, comorbidity burden, and multidrug-resistant infection further underscores the cumulative vulnerability of elderly males, who also demonstrated higher 30-day mortality and lower adequacy of empirical therapy.^3^,^5^ Although hospitalization rates were similar between sexes, the higher mortality among men suggests that initial disease severity or suboptimal empirical coverage may partially explain these differences.^9^

Importantly, when adjusting for sepsis and septic shock at presentation, male sex remained independently associated with multidrug-resistant infection, whereas disease severity emerged as the strongest determinant of short-term mortality. This indicates that part of the observed sex difference in outcomes is mediated by greater initial infection severity among men, consistent with prior sepsis cohorts. Integrating severity assessment into risk stratification models may, therefore, refine early management decisions and improve outcome prediction. These observations are consistent with the additional multivariable analyses performed in our cohort, which showed that infection severity (sepsis or septic shock) significantly contributed to the disparity in short-term outcomes between sexes. This underscores the importance of early recognition of sepsis and risk-adapted management strategies, particularly among high-risk subgroups, such as older male patients. Incorporating both severity and sex-based risk factors into ED decision-making may refine empirical treatment selection, support more personalized care pathways, and potentially reduce treatment failure. Future prospective studies should evaluate the impact of such personalized, risk-adapted approaches on patient-centered outcomes and antimicrobial resistance trends.

Our results are consistent with prior inpatient and community-based studies identifying male sex, recent hospitalization, and antibiotic exposure as key predictors of multidrug-resistant uropathogens.^1^–^3^ However, our study extends these findings to the ED population, where rapid empirical decision-making is critical and microbiological results are often unavailable at presentation. The identification of sex and age as readily available clinical predictors can, therefore, support more precise risk stratification and early optimization of antibiotic therapy in acute care. Emerging ED tools (eg, the González del Castillo multidrug-resistant risk scale) may help operationalize this approach when combined with local antibiograms.^10^,^11^

Incorporating these demographic and clinical factors into ED triage algorithms and empirical treatment protocols may enhance antimicrobial stewardship and improve short-term outcomes.^1^,^4^ This aligns with the findings of the recent UTILY stewardship substudy (Macera et al, in press), which demonstrated that persuasive antimicrobial stewardship interventions can significantly optimize antibiotic use in ED patients with UTI.^15^ In particular, a tailored therapeutic strategy—including broader initial empirical coverage for elderly male patients with multiple comorbidities, followed by timely de-escalation once culture results are available—could balance effectiveness and antimicrobial stewardship goals. Evidence from ED sepsis cohorts suggests that choosing agents with ≥ 70% on-arrival susceptibility based on institutional antibiograms is associated with lower in-hospital and 30-day mortality and shorter intensive care unit stay; consensus guidance for UTI management similarly emphasizes risk-adapted empiric therapy and early review for de-escalation.^12^–^13^

## LIMITATIONS

This study has several limitations. Our cohort reflects ED presentations rather than the full spectrum of community UTIs. The lack of standardized microbiological sampling across centers and incomplete culture data in some cases may also affect pathogen distribution estimates. Additionally, details on antibiotic timing and dosage were unavailable. Nevertheless, the multicenter design, large sample size, and inclusion of consecutive ED patients strengthen the external validity and generalizability of the findings.

Although we used the CCI as a standardized measure of comorbidity burden, this index does not capture all factors associated with the risk of multidrug-resistant infections. In particular, variables such as cumulative antibiotic exposure, residence in long-term care facilities, or detailed healthcare contact history were not systematically collected and may represent additional confounders. Age was analyzed using a ≥ 65-year threshold, which is commonly used to define older adult populations. However, this approach may mask heterogeneity within the younger age group, and future studies could explore more granular age stratification.

Future prospective studies are warranted to validate these observations and to evaluate whether integrating sex- and age-based empirical therapy algorithms into emergency care pathways can improve early outcomes and reduce antimicrobial resistance in patients with urinary tract infection.^11^

## CONCLUSION

Patients presenting to the ED with urinary tract infections show distinct clinical and microbiological patterns influenced by both sex and age. Male and older patients were more likely to harbor multidrug-resistant pathogens and to experience worse short-term outcomes, and male sex remained an independent predictor of multidrug-resistant infection after adjustment for age and comorbidity. These findings highlight the need to incorporate sex- and age-based risk assessment into empirical antibiotic selection in the emergency setting, and may help identify patients at higher risk of multidrug-resistant infection and support risk-adapted empirical therapy. Such an approach may improve the adequacy of initial therapy and reduce adverse outcomes.

## Figures and Tables

**Figure 1:**
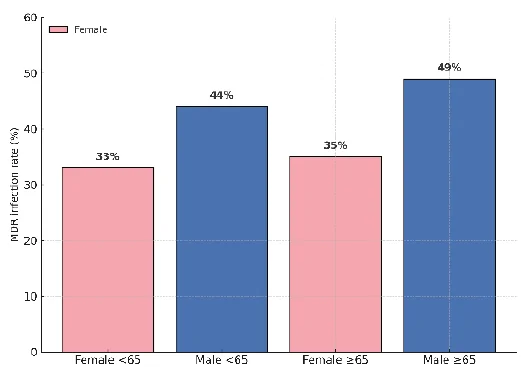
Multidrug-resistant infection rate by sex and age group among patients presenting to the emergency department with a urinary tract infection. *MDR*, multidrug-resistant.

**Figure 2:**
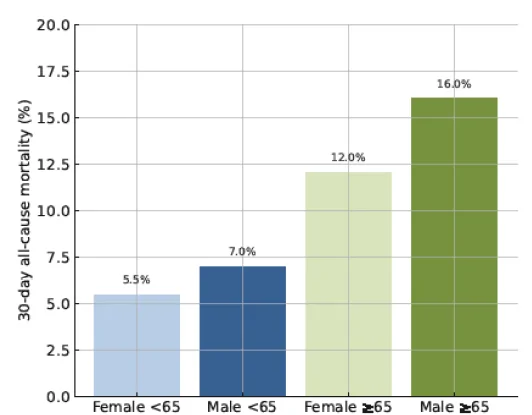
Thirty-day all-cause mortality by sex and age group among emergency department patients with urinary tract infection.

**Table 1 t1-wjem-27-1623:** Demographic and clinical characteristics by sex and age in a study evaluating sex-related differences in antimicrobial resistance and outcomes among adults with potentially multidrug-resistant urinary tract infections.

Variable	<65 yrs Female (n = 263)	<65 yrs Male (n = 224)	≥65 yrs Female (n = 257)	≥65 yrs Male (n = 277)	*P* (Sex overall)
Age, mean(SD)	59.1 (21.4)	63.1 (17.9)	67.1 (20.2)	74.1 (12.5)	< .001
Charlson Comorbidity Index, mean (SD)	1.60 (2.00)	2.11 (2.10)	2.10 (2.35)	2.92 (2.45)	.01
Multidrug-resistant infection, %	33.5	46.5	33.5	46.5	.02
Hospitalized, %	52.3	50.4	59.6	54.2	.09
Adequate empirical therapy, %	66.2	61.8	66.2	61.8	.10
7-day mortality, %	3.2	5.5	4.5	7.9	.05
30-day mortality, %	6.8	10.4	9.9	14.0	.05

* Data are presented as mean (standard deviation) or percentage (%). Comparisons between sexes were performed using the Mann–Whitney U test for continuous variables and the chi-square or Fisher exact test for categorical variables.

*SD*, standard deviation.

**Table 2:** Isolated pathogens by sex and age in a study evaluating sex-related differences in antimicrobial resistance and outcomes among adults with potentially multidrug-resistant urinary tract infections.

Pathogen	<65 yrs Male (%)	<65 yrs Female (%)	≥65 yrs Male (%)	≥65 yrs Female (%)
*Escherichia coli*	35.7	50.6	28.9	52.8
*Klebsiella spp.*	7.2	10.2	5.7	11.5
*Enterococcus spp.*	7.1	2.3	5.8	2.7
*Pseudomonas spp.*	2.7	0.4	2.2	0.8
*Proteus spp.*	3.1	1.9	2.5	1.9
*Other organisms*	44.2	34.6	54.9	34.1

The distribution of isolated pathogens differed significantly between sexes (χ^2^ test, *P* < .001), with *E coli* more frequently isolated in female patients and non-*E coli* pathogens, particularly *Enterococcus spp.*, more common among males. In contrast, no significant differences in pathogen distribution were observed between age groups (< 65 vs ≥ 65 years; *P* = .73).

**Table 3:** Outcomes, multidrug resistance, and empirical therapy adequacy by sex and age group in a study evaluating sex-related differences among adults with potentially multidrug-resistant urinary tract infections.

Variable	Female <65	Male <65	Female ≥65	Male ≥65	*P* (sex)	*P* (age)
Multidrug-resistant infection (%)	33.5	46.5	33.5	46.5	.02	.01
Hospitalization rate (%)	48.0	45.0	60.5	52.0	.08	<.01
7-day mortality (%)	2.0	3.5	6.5	9.0	.04	<.01
30-day mortality (%)	5.5	7.0	12.0	16.0	.03	<.01
Adequate empirical therapy (%)	69.0	64.0	63.0	59.0	.09	.12
Clinical response at 7 days (%)	88.0	80.0	85.0	75.0	.02	.03
